# Prevalence and associated factors of physical inactivity among adult diabetes mellitus patients in Felege Hiwot Referral Hospital, Bahir Dar, Northwest Ethiopia

**DOI:** 10.1038/s41598-022-26895-4

**Published:** 2023-01-04

**Authors:** Addis Enyew, Kalkidan Nigussie, Tewodros Mihrete, Musa Jemal, Shemsu kedir, Emana Alemu, Bekri Mohammed

**Affiliations:** 1 Department of Physiotherapy, School of Medicine, College of Medicine and Health Science, University of Gondar, Gondar, Ethiopia; 2 Department of Human Nutrition, Institute of Public Health, College of Medicine and Health Sciences, University of Gondar, Gondar, Ethiopia; 3 Department of Public Health, College of Medicine and Health Science, Werabe University, Werabe, Ethiopia; 4 Ethiopian Public Health Institute, Addis Ababa, Ethiopia

**Keywords:** Health care, Medical research

## Abstract

Physical inactivity has been viewed as an emerging public health problem in developing countries including Ethiopia. Diabetes mellitus (DM) is a group metabolic disorder characterized by chronic hyperglycemia resulting from defects in insulin secretion, function, or both. Its prevalence increases with changing lifestyles including physical inactivity across the globe. However, there is limited research, and not yet received attention in Ethiopia. This study aimed to assess the prevalence and associated factors of physical inactivity among adult diabetic patients in Felege Hiwot Referral Hospital, Bahir Dar, Northwest Ethiopia. An institutional-based cross-sectional study design was conducted among 308 participants from February to June 2018 at Felege Hiwot Referral Hospital. A face-to-face interview was conducted using a structured questionnaire by trained data collectors. Participants were selected through a systematic random sampling technique. Physical inactivity was assessed by the international physical activity questionnaire (IPAQ). Collected data were entered in Epi info version 7 and transferred to SPSS version 20 for analysis. A summary of descriptive statistics and multiple binary logistic regression analyses were computed to identify associated factors of physical inactivity among adult diabetic patients. P < 0.05 with 95% CI was considered statistically significant. The overall prevalence of physical inactivity among diabetic patients was 30.5% ( 95% CI: 22.8–33.5%). Gender (AOR = 1.5, 95% CI: 1.1, 3.62), Old age (AOR = 18.17, 95% CI: 22.7, 61.9) Residence (AOR = 4.24, 95% CI: 1,12,16.028), Low self-efficacy (AOR = 20.59, 95% CI: 10.598, 41.608), Poor attitude (AOR = 2.75, 95%CI: 1.44,5.28), and Lack of social support (AOR = 4.22, 95% CI: 1.28,4.07) were found significantly predictor factors of physical inactivity. The prevalence of physical inactivity in this study was high. Being female, old age, dwelling in an urban, having low efficacy, poor attitude, and lack of social support was greater risk factors for being physically inactive. Diabetic education should focus on engagement in physical activity by overcoming barriers to performing physical activity. Government and health professionals should emphasize that evidence-based physical activity important to change their attitudes and require reaching a consensus on social support by their families.

## Introduction

Diabetes mellitus (DM) is a group metabolic disorder characterized by chronic hyperglycemia resulting from a defect in insulin secretion, function, or both^1^. It can result in macro and microvascular diseases^1,2^. Its prevalence increases with changing lifestyles including physical inactivity across the globe^3^.

In 2014, World Health Organization (WHO) reported, that 422 million adults had DM, and expected to rise to 642 million by 2040^4^. According to International Diabetes Federation (IDF) 2015, 14.2 million adults had DM in sub-Saharan Africa. This report puts Ethiopia in the fourth position, having more than 1.3 million DM patients^5^.

In recent decades, studies have stated that physical activity (PA) is a key element in the management of DM^6–8^. It can contribute to reducing long-term or slowing the progression of existing DM-related complications^9^. Despite its many benefits, PA remains underutilized in self-care management, and the majority of the DM population in developing countries does not become physically active due to socio-demographic transition, rapid urbanization, and a sedentary lifestyle^10^.

Physical inactivity is a global pandemic and one of the most serious public health problems^11^. It is an independent risk factor for DM-related complications such as cardiovascular diseases, neuropathy, foot ulcer, retinopathy, and nephropathy^12^. Its prevalence continually rises from 30%- 60% both in developed and developing countries^10,13–15^. As per WHO 2010 report, physical inactivity is one of the fourth leading causes of global mortality and causes an estimated 3.2 million deaths worldwide. Of these 2.6 million were in low- and middle-income countries^16^.

Currently, physical inactivity is considered a serious public health problem and the fourth leading risk factor for global mortality^17^. Different studies reported that the prevalence of physical inactivity varies widely in both developed and developing countries, due to socio-demographic transition and rapid urbanization^2,18–21^.

A cross-sectional study conducted in New York, America showed that the prevalence of physical inactivity among DM patients was 60%^22^. In a similar study in Savant, Brazil, was 47.9%^20^. In another study conducted in Saul Paulo, Brazil was 62.5%^23^. The clinical cross-sectional study was carried out at the University of Valencia, Spain, and the prevalence of physical inactivity among DM patients was 35.7%^24^.

Another similar study conducted in Istanbul, Turkey showed that 39.5%, 51.9%, and 8.5% of respondents had low, moderate, and high physical activity levels respectively^25^. In contrast in Hamadan, Iran 25.9%, 57.5%, and 16.5% of the participant had low, moderate, and high physical activity levels respectively^2^. A similar study was conducted in Nepal and Manila, Philippines 43.3% and 31% had low physical activity levels respectively^26^.

A systematic review conducted in west African countries reported that the prevalence of physical inactivity in adult DM patients was 13%^18^. On the other hand, a cross-sectional study conducted in southwest Ogun state, Nigeria in 2004, on physical activity levels among type two DM indicated that 31% of the participant were inactive^14^. A similar study conducted in the same place in 2012 revealed reviled that 62% of the participants had low physical activity, 34% had moderate physical activity and only 4% had high physical activity^16^.

Literature reported that lack of local facilities, lack of social support, cost of exercise facilities, lack of time, fear of injured, and laziness could be factors for physical inactivity^21,27,28^. WHO recommended to all adults engage in a minimum of 75 min/week of vigorous and 150 min/week of moderate PA or achieve at least 600 metabolic equivalents (MET-minute) throughout a week. Where MET is the ratio of the rate of energy expended during an activity to the rate of energy expended at rest^9^.

Physical inactivity is commonly associated with sociodemographic (age, gender, marital status, education, occupation, income, residence, religion, type and duration of DM), psychological (self-efficacy and attitude), behavioral (smoking and alcohol consumption), environmental factors(lack of adequate facilities and lack of social support), anthropometric characteristics (body mass index, waist circumference, hip circumference) comorbidities of DM [hypertension, cardiovascular diseases and complication of DM]^2,4,12,13,19,26,29–33^.

Despite its known serious public health problem, physical inactivity has not yet received attention^34^ and there is also scarce data on the magnitude and determinant factors of physical inactivity among patients with DM in Ethiopia. Therefore, the Presence of well-documented data on physical inactivity among DM patients is crucial for intervention and research purposes. Thus, this study aimed to assess the prevalence and factors associated with physical inactivity among adult DM patients in Felege Hiwot referral hospital, Bahir Dar, Northwest Ethiopia.

## Methods

### Study design and setting

An institutional-based cross-sectional study design was conducted from February to June 2018.

The study was conducted at Felege Hiwot Referral Hospital, which is found in Bahir Dar city administration Northwest, Ethiopia. Bahir Dar is the capital city of the Amhara region and is located 550 km from Addis Ababa, the capital city of the country. Felege Hiwot Referral Hospital is a tertiary health care level hospital serving around 12 million population of Bahir Dar town and remote areas of Northwest, Ethiopia. Currently, it is the only governmental Hospital in the city and the regional referral hospital serving the population in the region as a referral center. It provides promotive, curative, and rehabilitative services to those populations. The reason to select this study area was that DM patient flow is high and the principal investigator noticed a lack of adequate recommended physical activity education for DM patients by healthcare professionals.

### Sample size and sampling procedure

The sample size was calculated by assuming a 5% margin of error, 95% confidence interval (alpha = 0.05), and 50% proportion of physical inactivity among DM patients since no study has been done in the study area with the same population. Based on this assumption, the sample size was calculated by using the single population proportion formula, and by considering a 10% non-response rate, the final sample size reached 308.

A systematic random sampling technique was used to select the study participants. The diabetic clinic provides its service two days per week (i.e. Monday and Tuesday); on average 170 DM patients were treated per week. Based on this, 1020 DM patients may be expected to follow up within six weeks data collection period. The sample interval was determined by dividing the expected number of DM patients (1020) during the course of the data collection period divided by the sample size (308) of the study which gave a sample interval of 4. Thus, every four patients were interviewed until the total sample size was reached. The first respondents were selected randomly with a lottery method.

### Data collection tools and procedures

Data was collected through face-to-face interviews, recorded from patient files, and measuring anthropometric characteristics. Body weight was measured by a weight scale without shoes. Height, waist, and hip circumferences were measured by using a non-elastic measuring tape. Waist circumference was taken at umbilicus level directly over the skin whereas hip circumference over the buttock area with clothing in a standing position^31^. As per WHO guidelines the cut-off point for waist and hip circumference ratio to the normal range for both men (< 0.95 cm) and women (< 0.85 cm)^21^**.**

Four data collectors (two physiotherapists and two nurses) were trained by the questionnaire investigator for two days before data collection. The questionnaire was pretested on 5%^15^ of the sample among DM patients who were not included in the main study area, in Gondar university hospital to detect potential problems, unanticipated and interpretations as well as to amend cultural issues to each question. The questionnaire was modified and corrected based on the pretest result. Furthermore, on each data collection day, the collected data were checked and reviewed to completeness by the main investigator.

### Operational definition

#### Physical inactivity

Is those respondents who will not achieve WHO recommendations of total physical activity (level less than 600METs-minute/week) or reported practicing no physical activities will be classified as inactive. Whereas those who meet this criterion(> 600METs-minutes/week) will be classified as physically active^21^.

### Body mass index (BMI)

A person's weight in kilograms (kg) divided by his or her height in meters squared (m2). Based on WHO’s 2004, BMI classification considered participants who have BMI (kg/m^2^) < 18.5, 18.5–24.9, 25–29.9 and > 30 kg/m^2^ will be classified as underweight, normal range, overweight, and obese respectively^32^.

### Cigarette smoking

Those who smoke any tobacco products daily will be considered tobacco users^33^.

### Alcohol drinking

Those who consume any type of alcohol 2 or more bottles daily will be considered alcohol users^34^.

### Good attitude

Those respondents who scored equal to or above the mean value of attitude towards physical activity score^35^.

### Poor attitude

Those respondents who scored below the mean value of attitude towards physical activity score^35^.

### High self-efficacy

Those respondents who scored equal to or above the mean value of self-efficacy towards physical activity score^36^.

### Low self-efficacy

Those respondents who scored below the mean value of self-efficacy towards physical activity score^36^.

### Lack of social support

Those participants who have no family, friends, or peer support to do physical activity^37^.

### Lack of adequate facilities

Facilities not available to perform physical activity^37^.

### Waist–hip-ratio (WHR)

Person’s waist circumference divided by hip circumference. Based on WHO 2008, convened participants who have in the normal range for both men (< 0.95 cm) and women (< 0.85 cm)^38^.

### Measurement

A structured questionnaire was prepared to collect sociodemographic information, behavioral, psychological, and environmental factors. Comorbid factors and DM-related complications were recorded from the patient’s files. Physical inactivity was assessed by the international physical activity questionnaire (IPAQ). It assesses the frequency (days) and duration (hours or minutes) of vigorous-intensity, moderate-intensity, walking, and sitting activities during the last seven days^39^.WHO assigned a value of 8METs, 4METs, and 3.3 METs for the time spent in vigorous, moderate, and walking activities respectively. As per WHO guidelines, physical activity was calculated as the total MET-minute/week.

Walking MET-minutes/week = 3.3 × walking minutes × walking days, Moderate MET-minutes/week = 4.0 × moderate-intensity activity minutes × moderate days, Vigorous MET-minutes/week = 8.0 × vigorous-intensity activity minutes × vigorous-intensity days.

A combined total physical activity MET-min/week could be computed as the sum of Walking + Moderate + Vigorous MET-min/week scores. Then participants were classified as inactive if the participants were scored less than 600 MET whereas the participants classified in to physically active if the participants were scored greater than 600 MET per week^40^. The questionnaire was translated from the English version to the Amharic version by native Amharic speakers and translated back to English to insure its contingency.

### Data processing and analysis

Data were entered in EPI info version 7 and transferred to SPSS version 20. Missing values were checked and data cleaning was done. Descriptive statistics were calculated for demographic and economic factors, information related. Binary logistic regression analysis was applied and expressed as an odds ratio (OR) and 95% confidence interval (CI). All predictor variables that have an association in bivariable analysis with a *p*-value ≤ 0.25^41^ were entered into a multivariable logistic regression model to identify independent factors associated with dependent variables by controlling the effect of confounders. The model was built by entering method and its fitness was assessed by the likely hood ratio test and Hosmer–Lemeshow goodness-of-fit statistic test. The crude and adjusted odds ratios together with their corresponding 95% confidence intervals with P-value < 0.05 were computed and interpreted accordingly.

### Ethics approval and consent to participate

The study was conducted as per the Helsinki Declaration for biomedical research. Ethical approval and clearance were obtained from the University of Gondar, College of Medicine and Health Science, Institutional Review Board wth a reference number of 211/2018. An ethical clearance letter was submitted to the Felege-Hiwot Referral Hospital and a permission letter was obtained. Informed written consent was taken from each Adult.

## Results

### Socio-demographic characteristics

Three hundred two study participants were involved with a response rate of 98%. Those six diabetic patients who were selected for a sample could not participate due to refusing to take the interview. Of the total respondents, the majority of them 174 (57.6%) were male, 235 (77.8%) were orthodox Christians and 88 (29.9%) were government employees. In terms of marital status, more than two third of respondents 210 (69.5%) with Diabetic Mellitus were married and 38 (2.6%) were widowed. Occupational status of participants with DM, among the total respondents 88 (29.1%) were government employees, and 72(23.8%) were housewives.

Regarding age distribution, the mean and standard deviation of participants was 49.4 ± 10.5. As to the type of Diabetic Mellitus, 221 (73.2%) were type two and 176 (58.3%) of participants were less than the seven-year duration of DM. Likewise, the median monthly income of respondents was 3540 ETB (≥ 3000 ETB) and 101 (33.4%) of the participant attended college/university level (Table 1).

**Table 1:** Socio-demographic characteristics of study participants at Felege Hiwot Referral Hospital, Bahir Dar, Northwest Ethiopia, 2018 (n = 302)*.*

Variable	Frequency	Percentage (%)
**Gender**		
Male	174	57.6
Female	128	42.4
**Age in years**		
18–39	52	17.2
40–49	92	30.5
50–59	105	34.8
≥ 60	53	17.5
**Religion**		
Orthodox Christians	235	77.8
Muslim	52	17.2
Protestant	15	5.0
**Educational status**		
No formal education	84	22.8
Primary education	50	16.6
Secondary education	67	22.2
College and above	101	33.4
**Type of DM**		
Type one	81	26.8
Type two	221	73.2
**Duration of DM**		
< 7 years	176	58.3
≥ 7 years	126	41.7
**Monthly income**		
< 1000 ETB	46	15.2
1000–1999 ETB	17	5.6
2000–2999 ETB	51	16.9
≥ 3000 ETB	188	62.3

### Anthropometric and behavioral characteristics

Thirty-six participants (11.9%) declared that they were smoking cigarettes daily and 22 (7.3%) of them were also alcohol consumers. Most of the participants 145 (48.0%) were in the normal weight category on BMI whereas 120 (39.7%) and 37 (12.3%) were overweight and obese respectively. Regarding their central obesity, more than half of the participants 167 (55.3%) were in a normal category on WHR (Table 2).

**Table 2:** Anthropometric measurements and behavioral characteristics of study participant at Felege Hiwot Referral Hospital, Bahir Dar, Northwest Ethiopia, 2018(n = 302).

Variable	Frequency	Percentage (%)
**Smoking status**		
Yes	36	11.9
No	266	88.1
**Alcohol consumption**		
Yes	22	7.3
No	280	92.7
**Body mass index**		
18.5–24.9	145	48.0
25–29.9	120	39.7
≥ 30	37	12.3
**Waist hip ratio**		
Normal	167	55.3
Obese	135	44.7

### Psychological and environmental factor characteristics

Of the study participants, about 178 (58.9%) respondents had high self-efficacy, and more than two third of respondents 202 (66.9%) had a good attitude toward physical activity. Regarding the environmental factors, the majority of participants 233 (77.2%) reported that they lack adequate facilities to do physical activity, as well as 245 (81.1%) of them, had a lack of social support (Table 3).

**Table 3:** Psychological and Environmental characteristics of study participants at Felege Hiwot Referral Hospital, Bahir Dar, Northwest Ethiopia, 2018(n = 302).

Variables	Frequency	Percentage (%)
**Self-efficacy**		
High	178	58.9
Low	124	41.1
**Attitude**		
Good	202	66.9
Poor	100	33.1
**Adequate facilities**		
Yes	69	22.8
No	233	77.2
**Safe side walks**		
Yes	254	84.1
No	48	15.9
**Safe work environment to do PA**		
Yes	109	36.1
No	193	63.9
**Social support**		
Yes	57	18.9
No	245	81.1

### Comorbid factor characteristics

Of the total participants, 13 (4.3%) were suffering from cardiovascular diseases. Of these, 12 (4.0%) were diagnosed with coronary heart disease. As to blood pressure, 79 (26.2%) participants had increased blood pressure. Concerning their diabetic-related complications, 79 (26.2%) of respondents had different diabetic-related complications. Among those, 44 (14.6%) had retinopathy (Table 4).

**Table 4:** Comorbid factor characteristics of study participants at Felege Hiwot Referral Hospital, Bahir Dar, Northwest Ethiopia, 2018 (n = 302).

Variables	Frequency	Percentage (%)
**Cardiovascular diseases**		
Yes	13	4.3
No	289	95.7
**Hypertension**		
Yes	79	26.2
No	223	73.8
**DM complication**		
Neuropathy	35	11.6
Retinopathy	44	14.6
Diabetic foot ulcer	13	4.3
Nephropathy	8	2.6

### Basic characteristics and physical inactivity

Physical inactivity was higher among female 49 (38.3%) participants compared with males 43 (24.7%) as well as urban dwellers 84 (34.3%) than rural 8 (14.0%). Regarding the educational status of the participants, the prevalence of physical inactivity was 16 (33.35%), 26 (40.0%), 33 (31.7%), and 17 (20.0%) for those who have no formal education, primary education, secondary education and participants who were college and above category respectively (Table 5).

**Table 5:** Socio-demographic characteristics by physical inactivity study participants at Felege Hiwot Referral Hospital, Bahir Dar Ethiopia, 2018(n = 302).

Variable	Inactive N (%)	Active N (%)
**Gender**		
Male	43 (24.7%)	131 (75.3%)
Female	49 (38.3%)	79 (61.7%)
**Age**		
18–39	2 (3.8%)	50 (96.2%)
40–49	8 (8.7%)	84 (91.3%)
50–59	37 (35.2%)	68 (64.8%)
≥ 60	45 (84.9)	8 (15.1%)
**Religion**		
Orthodox Christian	58 (24.7%)	177 (75.3%)
Muslim	29 (55.8%)	23 (44.2%)
Protestant	5 (33.3%)	10 (66.7%)
**Marital status**		
Married	53(25.2%)	157(74.8%)
Divorced	1(6.7%)	14(93.3%)
Separated	33(31.7%)	71(68.3%)
Widowed	35(92.1%)	3(7.9%)
Educational status		
No formal education	36 (42.9%)	48 (57.1%)
Primary education	17 (34.0%)	33 (66.0%)
Secondary education	20 (29.9%)	47 (70.1%)
College and above	19 (18.8%)	82 (81.2%)
**Occupation**		
Farmer	5 (14.7%)	29 (85.3%)
Merchant Private employee	5 (14.3%) 2 (6.1%)	30 (85.7%) 31 (93.9%)
Government employee	14 (15.9%)	74 (84.1%)
House wife	38 (52.8%)	34 (47.2%)
Other	28 (70.0%)	12 (30.0%)
**Place of residency**		
Urban	84 (34.3%)	161 (65.7%)
Rural	8 (14.0%)	49 (86.0%)
**Type of DM**		
Type one	13 (16.0%)	68 (84.0%)
Type two	79 (35.7%)	142 (64.3%)
**Duration of DM**		
< 7 year	28 (15.9%)	148 (84.1%)
≥ 7 year	64 (50.8%)	62 (49.2%)
**Monthly income**		
< 1000 ETB	9(19.6%)	37(80.4%)
1000–1999 ETB	5 (29.4%)	12 (70.6%)
2000–2999 ETB	9 (17.6%)	42 (82.4%)
≥ 3000 ETB	69 (36.7%)	119 (63.3%)

### Prevalence of physical inactivity among DM patients

**Figure 1:**
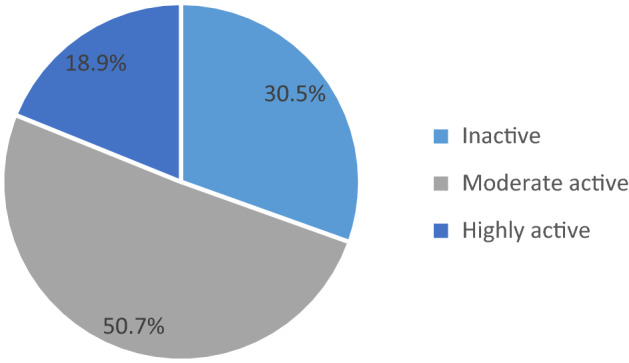
The prevalence of physical inactivity among participants with diabetic patient at Felege Hiwot Referral Hospital, Bahir Dar Ethiopia, 2018 (n = 302).

The overall prevalence of physical inactivity among participants with adult DM was 30.5% (95% CI: 22.8–33.5%) when a total metabolic equivalent which is a physical activity measurement cut of point 600 MET-minute/week was used. In contrast, 210 (69.5%) participants fulfilled the criteria of the physical activity category which was 600 MET-minute/week and above. Among the active group, the majority of the respondent 153 (50.7%) were performing moderate physical activity while only 57 (18.9%) were performing vigorous physical activity (Fig. 1).

### Factors associated with physical inactivity among DM patients

After controlling potential confounders variables such as gender, old age, place of residency, self-efficacy, attitude towards physical activity, lack of social support, and retinopathy were significantly associated with physical inactivity among diabetic patients (p < 0.05).

Diabetic patients who were female participants were 1.5 times more likely to be physically inactive (AOR = 1.5, 95% CI: 0.64, 3.62) than males. As well as those who are residing in urban areas were 4.24 more likely to be physically inactive (AOR = 4.24, 95% CI: 1.12, 16.02) compared with participants residing in rural areas. Regarding the age of participants, DM patients whose age is 60 years and above were 18.169 times more likely to be physically inactive (AOR = 18.16, 95% CI: 22.74, 61.95) compared with the younger age group.

Regarding self-efficacy and attitudes of participants toward physical activity, those respondents with low self-efficacy were 20.59 times more likely to be physically inactive (AOR = 20.59, 95% CI: 10.59, 41.60) than the high self-efficacy group. In addition, those who had poor attitudes also are 2.753 times more likely to be physically inactive (AOR = 2.75, 95%CI: 1.44, 5.28) than those who had a good attitude toward physical activity.

Individuals who lack social support were 4.22 times more likely to be physically inactive (AOR = 4.22, 95% CI: 1.28, 4.07) compared with those who have good social support (Table 6).

**Table 6:** Factors associated with physical inactivity on bivariate and multivariate logistic regression analysis of study participants at Felege Hiwot Referral Hospital, Bahir Dar Ethiopia, 2018 (n = 302)*.*

Variables	Physical Inactivity		OR 95% CI		P. value
	Yes	No	COR(95% CI)	AOR(95% CI)	
**Gender**					
Male	43	131	1	1	
Female	49	79	1.29 (1.15, 3.10)	1.51 (0.64, 3.62)	P = 0.039
**Residence**					
Rural	8	49	1	1	
Urban	84	161	3.19 (1.44, 7.05)	4.24 (1.12, 16.028)	P = 0.033
**Age**					
18–39	2	50	1	1	
40–49	8	84	2.18 (0.48, 11.65)	2.38 (0.48, 11.65)	P = 0.052
50–59	37	68	3.60 (1.13, 59.10)	7.83 (1.73, 25.32)	P = 0.043
≥ 60	45	8	14.62 (12.36, 17.19)	18.16 (22.74, 61.95)	P < 0.01
**Attitude**					
Good	39	163	1	1	
Poor	53	47	4.71 (2.78, 7.97)	2.75 (1.44, 5.28)	P < 0.01
**Social support**					
Yes	31	26	1	1	
No	80	153	2.48 (1.26, 4.89)	3.3 (1.59, 6.87)	P < 0.01
**DM complication**					
Retinopathy	38	6	3.92 (0.61, 5.54)	5.13 (0.15, 6.35)	P < 0.01

## Discussion

Physical inactivity is continually rising in many developing countries like Ethiopia and has been considered the fourth leading cause of global mortality^16^. Despite its huge negative impact, little is known about physical inactivity in patients with DM in Ethiopia particularly study area. Therefore, this study sought to assess the prevalence and associated factors of physical inactivity among adult DM patients in Felege Hiwot Referral Hospital North West Ethiopia.

This study found that the prevalence of physical inactivity among adult DM patients was 30.5% when the total metabolic equivalent cut-off point score (< 600 MET- minute/week) as per WHO guidelines, the international physical activity questionnaire (IPAQ) measuring tool was used^42^. Outdoor walking is the most commonly reported physical activity among the respondents. It might be due to the cost of joining Gym and exercise facilities, fear of being injured by practicing high-intensity activities, and self-belief like being embarrassed to wear sportswear, especially in old age and women.

This study’s prevalence was comparable with a similar study conducted in Nigeria found out relatively similar prevalence which was 31%^13^. It was also compared with the 31% prevalence reported from Manila, Philippines with a similar tool IPAQ used. This similarity might be due to similar methodology and measuring tools used^26^.

Prevalence of physical inactivity in this study was lower than in institutional based cross sectional studies conducted in New York, America (40%), Brazil (47.9%), Spain (35.7%), and Istanbul, Turkey (39.5%)^23–25^. The discrepancy between these studies may be due to methodological differences like sample size and study period. Another possible reason might be the socio-economic pattern means the majority of work-related activities changed to computerization and increased car ownership may reduce the chance to perform physical activity in those study participants while the majority of the respondents in our study expressed the long working hours represent the largest component of physical activity^43^.

In the study conducted in Saulo, Brazil, the prevalence of physical inactivity was 17.5%^20^. The possible reason for this difference might be the sample size and study participants. The Brazilian study includes only the urban population and unlike Ethiopia, the Brazilian federal government developed and disseminated national physical activity guidelines to on emphasize physical activity could be another reason^44^.

This study revealed that women participants were 1.5 times more likely to be inactive than male diabetic patients. This finding was supported by previous studies^14,21,45,46^. This is might be due to the women in developing countries like Ethiopia, commonly participating in light-intensity household chores activities such as housekeeping, carrying the babies, and food preparation activities which are routine daily activities. In urban areas these daily household activities commonly are carried out by domestic helpers whereas men are tending to participate vigorous activities like playing a sport, running, and cycling.

Furthermore, in this study, aged 60 and above was more likely to be physically inactive compared to younger age. This finding is in line with the studies conducted in Lebanon, Srilanka and Barcelona^12,14,24^, there is a positive significant association between an increase in age and decreased physical activity level on DM patients. The possible reason of this might be participants of this age group retired person and may be associated with a sedentary life style after retirement. Furthermore, older people are reluctant to do physical activity due to their perception to that DM weakened their body and causing them to have some demotivation effect to perform regular physical activity and feeling tiredness.

Moreover, urban residency was more likely to be physically inactive compare to rural residence. This finding is in line with previous studies done in Nigeria, Brazil and Barcelona^13,20,24^ showed that physical inactivity among those participants residing in urban (34.3%) area was higher than rural (14.0%) population. This might be due to participants who residing in urban area may distracted by television long time, computerization and mostly use passive mode of transport while majority of those rural participants perform high demanding work activity like farming and fishing as well as lack public transport access may a factor.

Regarding to the psychological attributes, this study agreed with other previous studies^2,21,29^ revealed that low efficacy and poor attitude towards physical activity were a major predictors of physical inactivity among adult DM patients. In this regard participants who had low efficacy towards physical activity were 20.59 times more likely physically inactive compared with those high efficacies. Furthermore, participants who had poor attitude towards physical activity also 2.75 times more likely risk of physically inactive compared with those who had a good attitude towards physical activity. This may arise by wrong belies and poor knowledge about benefit of physical activity as well as inadequate educational intervention and adapts self-care behavior, particularly physical activity for better management of the diseases.

Concerning to the environmental factors, our study revealed that lack of social support have statistical positive significant with physical inactivity on diabetic patients. Those participants who had lack of social support (family, peers, and friends) were 4.22 times more likely to be inactive compared with those who had social support. This study was supported by cross sectional studies conducted in Hamadan and UK^2,28^. This is may be due to that families or friends of participants who had no social support may have no money to pay in exercise facilities, recreational facilities which needs to pay money and may have also poor knowledge about the benefit of physical activity.

Unlike other studies so far^17,24,47^ behavioral factors such as smoking cigarettes, alcohol consumption and anthropometric measurements like general obesity (BMI) and central obesity (WHR) were not significant associated in our study. This might be because of low prevalence in smoking and alcohol users in our study. As well as those who were overweight, obese and higher central obesity individuals being motivated to engage physical activity in order to have weight loss.

### Limitation of the study

To the best of investigators knowledge, this is the first study that assessed the magnitude and determinant factors among DM patients in Ethiopia. Due to this, it was impossible to compare results with previous findings. This can be a limitation of this study. In addition, physical inactivity was assessed by using Questionnaires (subjective method approach) which inherited to recall bias. However, this instrument is internationally accepted reliable, and validated tool for cross-sectional study worldwide^42^.

Despite these limitations, it is a pioneer study in the country and adds to the limited body of evidence by providing useful data on the prevalence of physical inactivity among adult diabetic patients in Ethiopia, particularly in this study area.

## Conclusion

Overall this study found that almost one-third (30.5%) of participants with DM did not practice recommended physical activity level in Felege Hiwot Referral Hospital Bahir Dar, Northwest Ethiopia. Outdoor walking was the most commonly reported practiced physical activity.

Gender, older age, place of residence, low self-efficacy, poor attitude towards physical activity, lack of social support, and diabetic related complication (retinopathy) were significantly associated with physical inactivity among adult diabetic patients. Diabetic education should focus on engagement in physical activity by overcoming barriers to performing physical activity. Government and health professionals should empasize evidence based physical activity important to change their attitude and require to reach consensus on social support by their family.

## Data Availability

The datasets used and/or analyzed during the current study are available from the corresponding author based on reasonable request.
